# Descriptive Analysis of Patients Treated with Diroximel Fumarate and Dimethyl Fumarate—A Real-Life Experience

**DOI:** 10.3390/jpm15010012

**Published:** 2024-12-31

**Authors:** Marina Blanco-Ruiz, Belén Sánchez-Rodríguez, Maria Luisa Ruiz-Franco, Emilio Molina Cuadrado, Francisco Sierra García, Carmen Muñoz Fernández

**Affiliations:** 1 Multiple Sclerosis Team, Neurology Service, Hospital Universitario Torrecardenas, 04009 Almería, Spain; marial.ruiz.franco.sspa@juntadeandalucia.es (M.L.R.-F.); carmen.munoz.fernandez.sspa@juntadeandalucia.es (C.M.F.); 2 Hospital Pharmacy Service, Hospital Universitario Torrecardenas, 04009 Almería, Spain; belen.sanchez.rodriguez.sspa@juntadeandalucia.es (B.S.-R.); emilio.molina.sspa@juntadeandalucia.es (E.M.C.); francisco.sierra.sspa@juntadeandalucia.es (F.S.G.)

**Keywords:** multiple sclerosis, lymphocytes, diroximel fumarate, dimethil fumarate, side effects

## Abstract

Background: Dimethyl fumarate (DMF) and diroximel fumarate (DRF) are two treatments used for multiple sclerosis (MS) that have been shown to be effective in controlling MS patients. DRF was introduced in 2019 with the aim of decreasing the gastrointestinal side effects caused by DMF. Few real-life studies verify the data provided in the clinical trials. Methods: A retrospective descriptive study was conducted on MS patients at the Hospital Universitario Torrecárdenas under treatment with DRF and DMF. Demographic, clinical, and analytical variables were studied and compared between groups. Results: A total of 60 patients were recruited, 30 with each treatment, observing similar baseline characteristics. Fewer gastrointestinal (GI) effects were observed in the DRF group, while more infections were detected in the DMF group. We recorded lower levels in the DRF group, with four cases of moderate-severe lymphopenia in the DRF group vs. none in the DMF group. In addition, we observed a decrease in lymphocytes after the change from DMF to DRF in patients with a change. Conclusions: Our real-life analysis of patients treated with DMF or DRF supports several studies’ findings regarding decreased GI side effects with DRF vs. DMF without decreasing efficacy. However, our data show a greater reduction in lymphocytes in patients with DRF compared to DMF, so more studies are necessary.

## 1. Introduction

Dimethyl fumarate (DMF) and diroximel fumarate (DRF) are two treatments used in multiple sclerosis (MS), an autoimmune disease of the central nervous system that affects millions of people worldwide [1,2]. Both compounds have been shown to be effective in reducing inflammatory activity and progression of flare-dependent disability in MS patients [3,4].

DMF, marketed under Tecfidera [5], was approved by the European Medicines Agency (EMA) in 2013 and reached the Spanish market that same year. It functions as an immune system modulator, although its precise mechanism of action in MS is not yet fully understood. It is believed to act by activating antioxidant and anti-inflammatory pathways, which reduces the autoimmune response that characterizes the disease [6,7].

On the other hand, DRF, marketed as Vumerity [8], is a delayed-acting form of fumarate developed to reduce the GI side effects associated with the use of dimethyl fumarate [9]. These GI symptoms mainly consist of recurrent diarrhea and constant gastric discomfort. While these symptoms are not severe and do not usually require hospitalization, they are often very disruptive to patients’ daily lives, so a new drug to reduce them was needed. It was approved by the FDA in the United States in 2019 and subsequently by the EMA in 2020, reaching the Spanish market that same year. Like DMF, DRF is believed to exert its action primarily through modulation of the immune system, albeit with an enhanced side effect profile.

Both drugs cause, as a consequence of their mechanism of action, a decline in lymphocytes which is not always constant but is of essential concern when monitoring, since it can lead to a higher probability of developing opportunistic infections and progressive multifocal leukopathy.

Few studies compare these drugs, particularly the levels of lymphopenia produced, which are well known with DMF but under-explored with DRF. Considering the importance of medical research and evidence-based conclusions, we conducted this study.

## 2. Materials and Methods

### 2.1. Ethical Aspects

The study followed the ethical principles agreed upon in the Declaration of Helsinki [10] and received a favorable certificate from the corresponding Ethics Committee. The protocol code is DMF001 and the internal validation code is 63/2024, dated the 8 July 2024.

### 2.2. Patients’ Selections

This is a retrospective descriptive study on patients diagnosed with Relapsing–Remitting Multiple Sclerosis (RRMS) according to the 2017 McDonald criteria, treated with DMF at the Hospital Universitario Torrecardenas. Thirty patients undergoing treatment with DRF were selected. An equal number of cases (N = 30) were chosen from patients who transitioned to DMF after previously receiving a different treatment. Specifically, these patients began DMF treatment between 2019 and 2022 and are still on the drug. Data were extracted from the medical records collected in the HUT multiple sclerosis database.

### 2.3. Data Collection

Variables from the regular follow-ups of the patients were collected at the demographic, clinical, and analytical levels. Regarding demographic variables, we collected data on sex, age, and time of disease duration. Regarding clinical variables, we collected the Expanded Disability Status Scale (EDSS) score and the appearance of clinical or radiological flares during follow-up. We also wanted to study the safety of the drug, and, for this purpose, we reviewed the use of antibiotics required by each patient, recorded as “infections”. As for the analytical variables, we focused mainly on lymphopenia. We measured lymphocyte levels, taken from a regular blood test, using quantitative variables (×10^3^/µL); categorically, mild was <1000 lymphocytes, moderate was between 500 and 1000, and severe was <500 lymphocytes.

### 2.4. Statistical Analysis

Initially, a descriptive analysis of the sociodemographic and clinical status of each patient was carried out. Quantitative variables were expressed as the mean and standard deviation or the median, accompanied by the interquartile range. Qualitative variables were represented by frequency and percentage. The normality of quantitative variables was evaluated with the Kolmogorov–Smirnov or Shapiro–Wilk test.

A bivariate analysis was conducted to assess whether significant differences existed in the sociodemographic and clinical variables between the treatment groups. A Student’s *t*-test was used for quantitative variables when the data followed a normal distribution and a Mann–Whitney U test was applied for non-normally distributed data.

Subsequently, the lymphocyte levels were compared, distinguishing groups at different times using dependent comparisons and a Student’s *t*-test for dependent samples or a Wilcoxon’s Sign-Rank test based on the previous results of normality.

The results are presented, including the respective 95% confidence intervals. The calculations were conducted with R Statistical Software (v4.1.2; R Core Team 2021) and SPSS version 29 (IBM Inc., Armonk, NY, USA).

## 3. Results

### 3.1. Demographic Data

A total of 60 patients were analyzed, 30 of whom were treated with DRF and 30 of whom were undergoing treatment with DMF. Their basic demographic data were examined, including age, sex, and disability measured by the EDSS. The average age was 40, with a predominance of the female sex (65%) and an average EDSS of 1.50. The data specified by groups are described in Table 1.

### 3.2. Higher Incidence of Side Effects in DMF Treated Patients

Of the 30 patients treated with DRF, 20 patients (66.7%) had previously been treated with DMF but needed to change treatments due to intolerance. Nineteen of them suffered relevant side effects that did not improve with symptomatic treatment. Only two patients maintained these side effects when changing to DRF. Table 1 shows the side effects that patients experienced with each treatment. We observed 38% of patients with GI symptoms, with these symptoms being significantly higher in patients treated with DMF (*p* < 0.001). Notably, 37% of patients experienced flushings; the amount was also higher in patients treated with DMF, but not to a significant degree.

Regarding efficacy, four patients (6.7%) had flares that led to a change in treatment; one was treated with DRF and the other three were treated with DMF. No patients showed signs of progression that were independent of the detected flares.

### 3.3. Lymphopenia Detected in Patients Treated with DRF

We have also evaluated the immunosuppression caused by both drugs, analyzing lymphocytes one month and three months after starting treatment and finding lower mean levels in those with DRF at the first month of treatment, as shown in Table 2. However, when analyzing lymphopenia categorically, we can observe how two patients in the DRF group suffered moderate lymphopenia and another two suffered severe lymphopenia; nevertheless, we cannot identify these results in the DMF group.

**Figure 1:**
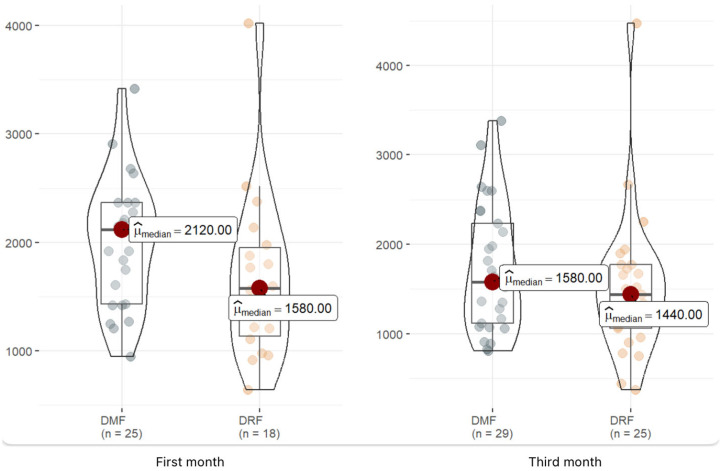
Graphical distribution of mean lymphocyte levels detected in patients in each group at the first and third month of treatment.

Figure 1 shows that the lymphocyte levels are lower in patients treated with DRF in the first and third months, but this difference is significant only in the first month.

**Figure 2:**
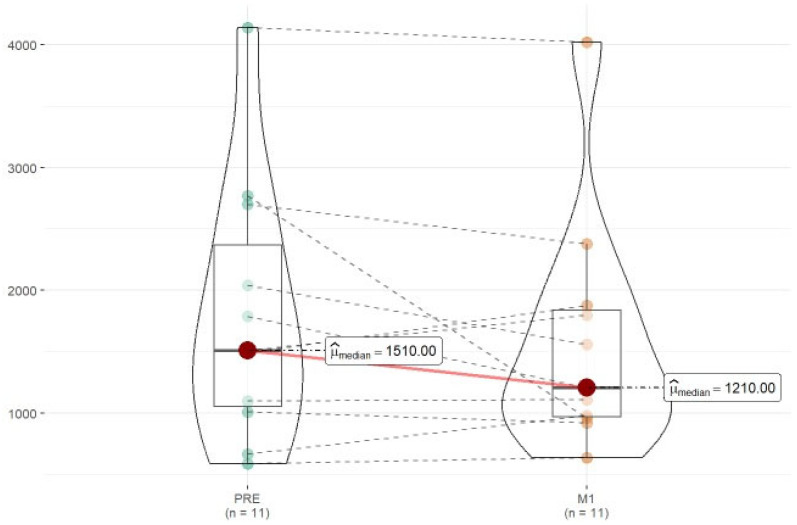
Graphical distribution of lymphocyte levels detected before the treatment changed from DMF to DRF and after one month of treatment, paired by cases.

Since two-thirds of the patients treated with DRF had previously been on DMF and were exposed to longer immunosuppression, we wanted to investigate whether the depletion of lymphocytes could be related to this. In order to achieve this, we analyzed lymphocyte levels in each patient who had been on DMF before switching to DRF, comparing their levels before and after the switch. As shown in Figure 2, there is indeed a decrease in lymphocyte levels after the change from DMF to DRF. DRF showed a greater decrease in levels compared to DMF, with lower mean lymphocyte levels in this drug and the appearance of moderate and severe lymphopenia rates, which did not occur with DMF.

On the other hand, we also analyzed the drugs with which patients had been treated before DMF and DRF to assess whether a more prolonged effect of immunosuppression could justify the drop in lymphocytes. In our sample, six patients in the DMF group were previously treated with other disease-modifying drugs (two with glatiramer acetate and four with interferons), while 17 patients in the DRF group received prior treatment (nine with glatiramer acetate and eight with interferons).

### 3.4. Higher Incidence of Infections in DMF Treated Patients

We also aimed to analyze the infections patients experienced with both drugs. To achieve this, we reviewed clinical records to identify those who had undergone antibiotic treatment since starting the medication. As shown in Table 1, 43% of patients (n = 26) had been treated with antibiotics, but patients treated with DMF (n = 17) required more antibiotics than those on DRF (n = 9).

## 4. Discussion

DMF is a widely known drug in MS clinics, as it has been presented for years as an effective drug for the control of relapsing–remitting diseases with a mild-to-moderate course. Nonetheless, one of the major problems with DMF has always been the side effects, especially GI features and flushings. In the aforementioned real-life study [11], 44% of patients had flushings and 35.7% had GI effects, of which about half remained after 3 months of treatment. Therefore, up to 44% of patients discontinued treatment because of several side effects.

This appears to be a solved obstacle in DRF treatment. A phase III comparative study [9] concluded that DRF showed a significantly lower incidence of GI side effects than DMF. In addition, Liseno et al. [12] conducted a study on 160 US patients, describing only 11% of GI side effects compared to the 44% previously reported in other real-life studies of DMF. These data are consistent with those observed in our sample, in which up to 63% presented GI symptoms with DMF, 90% of which resolved after switching to DRF.

It is important to note that the side effects of DMF began to manifest more consistently and intolerably after the introduction of generic DMF. However, due to challenges in tracking these patients, we cannot determine how many who switched from DMF to DRF had been using Tecfidera or a generic version.

Regarding efficacy, relapses were recorded in three patients on DMF, which required switching to a more highly effective drug, while only one patient on DRF had a relapse. However, to properly evaluate this difference, it is important to note that the DRF-treated patients started their treatment no more than two years ago, whereas the DMF group includes patients who began treatment as early as 2020, giving them a longer treatment duration and, consequently, a higher likelihood of experiencing an outbreak.

In the published studies [9,10], no increased risk of infection has been described with either drug. Although, in our sample, there is a higher use of antibiotics by patients treated with DMF, one possible explanation is the longer treatment duration for DMF patients. However, this is an important factor to consider in future studies.

Several studies have reported an increased risk of severe lymphopenia in those patients that present an over 21% decrease during the first three months of DMF treatment [13,14,15]. In a real-life study in Spain [11], 30% of patients presented with lymphopenia (<1000 lymphocytes). Although the effects of DMF have been thoroughly evaluated over its decade-long use, no comparable studies have been conducted for DRF. The results of the EVOLVE-MS-1 [16], an open-label, phase 3 study designed to evaluate the long-term safety and tolerability of DRF, have recently been published, analyzing lymphocyte levels in different groups of patients (those with de novo treatment, those previously treated with DMF and those previously treated with DRF). Their results show moderate levels of maintained lymphopenia, being 13.7% in the first group, 12.4% in the DMF group, and 16.4% in the DRF group.

In our sample, we observed a higher number of patients with lymphopenia and more severe lymphopenia in those treated with DRF compared to those treated with DMF. Two studies [17,18] have recently been published with similar results, indicating that patients who were stable with DMF experience greater lymphopenia when they switch to DRF. We have also been able to observe this in our patients where a decrease in lymphocytes was observed after the change from DMF to DRF, as shown in Figure 2, although it is not a significant difference.

As for the immunosuppressive treatments with which patients have been previously treated, it is true that they are higher in the DRF group than in the DMF group. Further research is needed to find out whether this may have an effect on the higher rates of lymphopenia found in the DRF group.

We acknowledge the limitations of our study, which are primarily due to its small sample size. However, the findings from our analysis are intriguing and pave the way for future research into the potential variability in efficacy and safety between these two drugs, particularly regarding lymphopenia.

## 5. Conclusions

Our real-life analysis of multiple sclerosis patients treated with DMF or DRF supports the findings in studies regarding decreased gastrointestinal side effects with DRF versus DMF without decreasing efficacy. However, our data show a greater reduction in lymphocytes in patients with DRF compared to DMF, so more studies are necessary to verify these results. This may have relevant implications when monitoring patients with these treatments, and it may even change vaccination and infection prevention strategies; therefore, in our opinion, it is important to clarify this aspect.

## Figures and Tables

**Table 1:** Demographic variables compared by treatment.

	N	Overall	Treatment		*p*-Value ^2^
			DRF, N = 30	DMF, N = 30	
Demography					
Sex (% Female)	60	39 (65%)	21 (70%)	18 (60%)	0.42
Age ^1^	60	40 (33, 46)	45 (38, 48)	36 (33, 41)	0.015
Side effects					
GI symptoms (%)	60	23 (38%)	2 (6.7%)	19 (63%)	<0.001
Flushing (%)	60	22 (37%)	9 (30%)	13 (43%)	0.28
Security					
Infections (%)	60	26 (43%)	9 (30%)	17 (57%)	0.037
Efficacy					
Outbreak (%)	60	4 (6.7%)	1 (3.3%)	3 (10%)	0.61
EDSS ^1^	60	1.50 (1.00, 2.00)	1.50 (1.00, 2.00)	1.25 (1.00, 2.00)	0.24
1 Median (IQR)					

^1^ Median (IQR). ^2^ Pearson’s Chi-squared test; Wilcoxon rank sum test; Fisher’s exact test.

**Table 2:** Lymphocyte levels and lymphopenia detected.

	Treatment Received				
	N	Overall	DRF	DMF	*p*-Value
Lymphocytes M1 ^1^ (×10^3^/µL)	43	1840 (1345, 2245)	1580 (1135, 1955)	2120 (1430, 2370)	0.058
Lymphocytes M3 ^1^ (×10^3^/µL)	54	1520 (1080, 1948)	1440 (1060, 1770)	1580 (1120, 2230)	0.21
Categories M1	43				0.42
Mild		42 (98%)	17 (94%)	25 (100%)	
Moderate		1 (2.3%)	1 (5.6%)	0 (0%)	
Severe		0 (0%)	0 (0%)	0 (0%)	
Categories M3	54				0.040
Mild		50 (93%)	21 (84%)	29 (100%)	
Moderate		2 (3.7%)	2 (8.0%)	0 (0%)	
Severe		2 (3.7%)	2 (8.0%)	0 (0%)	

M1: At the first month of treatment. M3: At the third month of treatment. ^1^ Median (IQR) or frequency (%).

## Data Availability

The data on the variables collected are publicly available at the following: https://doi.org/10.6084/m9.figshare.26207342.
